# NGF from pancreatic stellate cells induces pancreatic cancer proliferation and invasion by PI3K/AKT/GSK signal pathway

**DOI:** 10.1111/jcmm.15265

**Published:** 2020-04-15

**Authors:** Jie Jiang, Jun Bai, Tao Qin, Zheng Wang, Liang Han

**Affiliations:** ^1^ Department of Hepatobiliary Surgery First Affiliated Hospital Xi’an Jiaotong University Xi’an China; ^2^ Department of Medical Oncology Shaanxi Provincial People’s Hospital Xi’an China

**Keywords:** epithelial‐mesenchymal transition, NGF, pancreatic cancer, proliferation, TrkA

## Abstract

Pancreatic cancer (PC) is a continuously high lethal disease, and the tumour microenvironment plays a pivotal role during PC progression. Herein, we focus on that the Nerve growth factor (NGF)/Tropomyosin‐related kinase A (TrkA), in pancreatic stellate cells‐pancreatic cancer cells (PSCs‐PC cells) co‐culture system, influences PC proliferation and invasion. The model of PC cells and PSCs was directly co‐cultured in a no‐touch manner, using the Transwell as the co‐culture system. NGF and TrkA expression was measured in cultured system by real‐time PCR, immunofluorescence, Western blotting analysis or ELISA. Small interfering RNA transfection was used to regulate the expression of TrkA in PC cells. The promotion of cancer invasion was investigated using Matrigel Transwell assay. In our study, NGF/TrkA is overexpressed in PSCs‐PC cells co‐culture system and promotes the invasion and proliferation of PC cells. And the epithelial‐mesenchymal transition‐related genes are influenced by si‐TrkA. What's more, NGF/TrkA regulates the PC cell proliferation and invasion via activation of PI3K/AKT/GSK signalling. The present study demonstrated NGF/TrkA promoted the PC cell proliferation and invasion in the co‐culture system by the activation of the PI3K/AKT/GSK signal cascade, providing a potential therapeutic target for PC patients.

## 1 INTRODUCTION

Pancreatic cancer (PC) is a continuously high lethal disease that is diagnosed at a distant stage typically with a 5‐year survival rate which is very low, respectively.^1^ Despite there are some significant advances in the understanding of the pathological characteristics of PC, the mortality rate of this cancer has hardly changed, which indicates there are no substantial improvements in clinical treatment.^2^ The in‐depth study of the mechanism of PC invasion and migration provided important theoretical significance and clinical application value for improving the therapeutic effect of PC.^3^, ^4^

In many published studies, it has become apparent that the tumour microenvironment plays a pivotal role during pancreatic malignant progression.^5^, ^6^, ^7^ One of the hallmarks of PC is abundant and dense cellular matrix consisting of stromal cells and extracellular matrix (ECM).^8^ Pancreatic stellate cells (PSCs) are responsible for the extensive desmoplastic reaction which sometimes accounts for 50%‐80% of tumour volume.^9^ Because of this characteristic, general treatments for PC are less effective and easier resisted. PSCs are lipid‐storing cells existing in normal pancreatic tissue before activated by cytokines and other factors. PSCs can be activated by a variety of factors, including pro‐inflammatory factors, oxidative stress and factors in tumour microenvironment. Activated PSCs can be transformed into myofibroblast‐like cells which produce collagenous stroma.^10^ Also, some evidence shows that PSCs interact with PC cells through various active factors which promote tumour growth and progression.^5^ The interaction between PC cells and PSCs plays their roles through various active factors.^5^, ^11^

Nerve growth factor (NGF) is a member of the neurotrophin family. Numerous studies reveal that the high level of NGF in PC is closely correlated with tumour proliferation, tumour cell apoptosis and perineural invasion, especially.^12^, ^13^ Tropomyosin‐related kinase A (TrkA) is the high‐affinity receptor for NGF, in contrast with the low‐affinity p75 neurotrophin receptor (p75NTR).^14^ Phosphoinositide 3‐kinase (PI3K)/protein kinase B(AKT) signalling pathway, which is the downstream of NGF/TrkA, promotes cancer survival and proliferation.^15^, ^16^

Herein, we focus on that the high level of NGF in pancreatic stellate cells‐PC cells (PSCs‐PC cells) co‐culture system influences PC proliferation and epithelial‐mesenchymal transition (EMT). We built an indirect PSCs‐PC cells co‐culture system in vitro and used the model to explore the NGF/TrkA works in this system. The data showed that NGF/TrkA promotes PC proliferation and EMT by PI3K/Akt/GSK signal pathway.

## 2 MATERIALS AND METHODS

### 2.1 Cell culture and reagents

The human PC cell lines AsPc‐1 and Panc‐1 were obtained from the American Type Culture Collection and cultured in DMEM supplemented with 10% foetal bovine serum (FBS) and 1% antibiotics/antimycotics in a humidified 5% CO_2_ atmosphere at 37°C. Antibodies against MMP‐9, vimentin, E‐cadherin, NGF, TrkA, p‐AKT, AKT, GSK and p‐GSK were purchased from Abcam. Recombinant NGF was obtained from R&D Systems.

### 2.2 Real‐time PCR

Total RNA was extracted using TRIzol (Invitrogen), and cDNA was synthesized using a PrimeScript RT Reagent Kit (TaKaRa). The real‐time experiments were conducted on an iQ5 Multicolor Real‐Time PCR Detection System (Bio‐Rad) using a SYBR Green Real‐time PCR Master Mix (TaKaRa). The primers are shown in Table 1.

**Table 1:** Real‐time PCR primer sequence

Genes	Primer sequence
TrkA	P1: 5′‐GGT ACC AGC TCT CCAACA CTG AGG‐3′ P2: 5′‐CCA GA ACG TCC AGGTAA CTC GGT G‐3′
E‐cadherin	P1: 5′‐ACA GCC CCG CCT TAT GAT T‐3′ P2: 5′‐TCG GAA CCG CTT CCT TCA‐3′
Vimentin	P1:5′‐GAGAACTTTGCCGTTGAAGC‐3′ P2:5′‐GCTTCCTGTAGGTGGCAATC‐3′
NGF	P1: 5′‐AAGGCTTTGCCAAGGACG‐3′ P2: 5′‐GTGATGTTGCGGGTCTGC‐3′
GAPDH	P1: 5′‐ACCACAGTCCATGCCATCAC‐3′ P2: 5′‐TCCACCACCCTGTTGCTGTA‐3′

### 2.3 Western blotting analysis

Cells were lysed using a lysis buffer (50 mM Tris [pH 7.5], 150 mM NaCl, 1% NP 40, 0.5% sodium deoxycholate, 1 mM EDTA and 0.1% SDS) containing a protease inhibitor cocktail (Sigma‐Aldrich), and protein concentrations were measured with the DC Protein Assay (Bio‐Rad Laboratories, Inc). After separation on 7.5% SDS‐polyacrylamide gels, proteins were transferred to nitrocellulose membranes (Amersham Bioscience), which were then incubated with primary antibodies at 4°C overnight. After being washed 3 times with TBST, the membranes were incubated with horseradish peroxidase–conjugated secondary antibodies for 1 hour. Immunoreactive bands were visualized using an enhanced chemiluminescence kit (Millipore). Quantitative analysis was performed using Image‐Pro Plus 6.0 software (Media Cybernetics, Inc). The relative protein expression levels were normalized to GAPDH.

### 2.4 MTT assay

Cell proliferation rate was measured by MTT assays. The cells were seeded in 96‐well plates at a density of 1 × 10^4^ cells per well and incubated overnight in medium containing 10% FBS. The DMSO concentration was adjusted to 0.4%. The cells incubated in serum‐free medium were used as the control group. Following incubation for 12, 24 and 48 hours at 37°C, 20 μL of MTT solution (5 mg/mL in PBS) was added to each well, and the cells were incubated for an additional 4 hours at 37°C. Subsequently, 100 μL DMSO was added to each well at 37°C. The optical density (OD) value was determined using a spectrophotometer (Bio‐Rad Laboratories Inc) at 490 nm. The proliferation rate was defined as OD (cell plate)/OD (blank plate).

### 2.5 Co‐culture system for PC cells and PSCs

The fresh pancreas tissue (from the liver transplant donors in the First Affiliated Hospital of Xi'an Jiaotong University) was made as 1‐2 mm^3^, and the adherence of tissue and the morphology and quantity of cell were observed under a phase‐contrast microscope. All experimental protocols were approved by the Ethical Committee of the First Affiliated Hospital of Xi'an Jiaotong University, Xi'an, China. The PSCs began to grow 3‐day encompassment of the pancreas tissue and were isolated from pancreas. PSCs were subsequently seeded on 24‐well plates cultured in DMEM supplemented with 10% FBS and 1% antibiotics/antimycotics in a humidified 5% CO_2_ atmosphere at 37°C.

We propose a model in which the PC cells and PSCs were directly co‐cultured in a no‐touch manner. Using the Transwell as the co‐culture system, the upper chamber was inoculated with 1 mL of human PSC suspension (cell density 2 × 10^5^/mL). PC cells suspended in 1.2 mL (cell density 2 × 10^5^/mL) for the different groups were inoculated into the lower plate and cultured for 24 hours at 37°C in a 5% incubator.

### 2.6 Immunofluorescence

NGF was localized in PSCs by immunofluorescence. The prepared PSCs were washed 3 times with PBS and then fixed with 100 mL 4% paraformaldehyde in PBS. The cells were permeabilized in blocking buffer (0.1% Triton X‐100 or 0.1%‐0.5% saponin, 10% NGS, 100 mM PBS, pH 7.4) for 1 hour at room temperature and then incubated with primary antibodies overnight at 4°C. Next day, the slides were incubated with secondary antibody anti‐rabbit IgG FITC‐conjugated (1:100) (Invitrogen) at RT. The cellular localization of protein was studied with fluorescence microscope.

### 2.7 Migration experiment

The invasiveness of PC cells was assessed based on the invasion of cells through Matrigel‐coated Transwell inserts. Briefly, the upper surface of a filter (pore size, 8.0 μm; Millipore) was coated with basement membrane Matrigel (BD Biosciences). The cells were suspended in DMEM with 1% FBS. Cell suspensions (100 μL, 10 000 cells) were then added to the upper chamber. Next, 500 μL of DMEM containing 20% FBS was simultaneously placed into the lower chamber of the Transwell. The cells were allowed to migrate for 24 hours at 37°C. The non‐migrated cells were removed from the upper surface by scraping with a cotton swab. After incubation, the filter was fixed and stained with crystal violet. All cells that had migrated from the upper to the lower side of the filter were quantified using a light microscope by counting in 10 random microscope views.

### 2.8 ELISA

Conditioned medium obtained from the co‐culture system was collected at 24 hours after the treatments, centrifuged (1200 rpm) for 10 min and frozen at 80°C until analysed. The levels of secreted NGF were determined by an enzyme‐linked immunosorbent assay (ELISA) (R&D Systems) according to the manufacturer's instructions.

### 2.9 Transfection

Silencing of gene expression was achieved using siRNA technology. Tumour cells were transfected with TrkA siRNA. Cells were seeded into small dishes and transfected with 100 nmol/L SiRNA using Lipofectamine 2000 (Invitrogen) according to the manufacturer's instructions. The cells were used for further experiments 6 hours after transfection. Negative control siRNA (Ambion Inc) was used as a negative control.

### 2.10 Statistics

The analyses of the results were carried out using the spss statistical software package (version 16.0). The significance of the data was determined using Student's *t* test or ANOVA analysis. A value of *P* < .05 was considered to indicate a statistically significant difference. Data are representative of at least three independent experiments and are reported as means ± SD.

## 3 RESULTS

### 3.1 NGF is expressed in PSCs isolated from human pancreas

**Figure 1:**
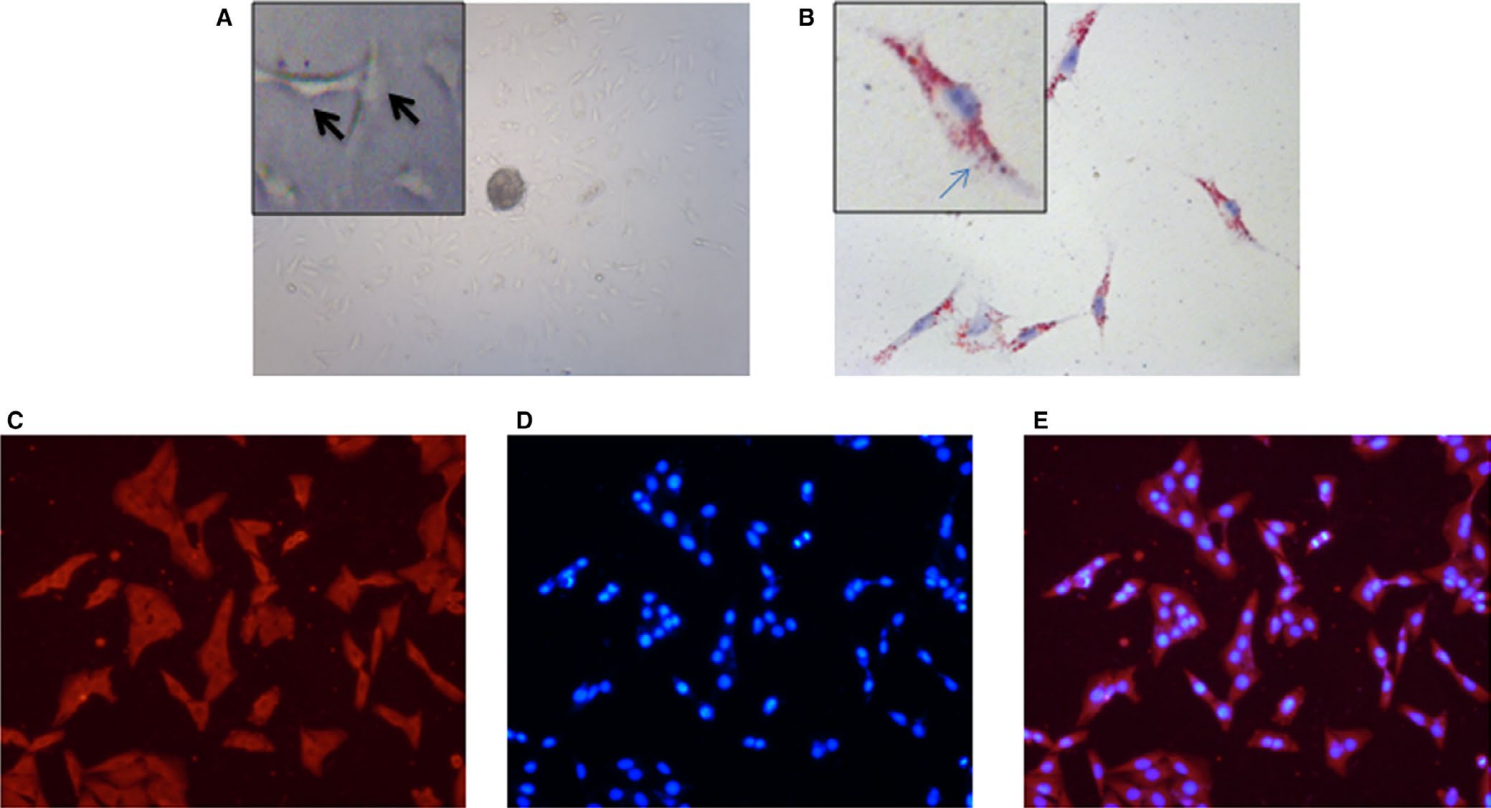
NGF is highly expressed in PSCs isolated from human pancreas. (A) Primary PSCs were isolated from human pancreas, and the adherence of tissue and the morphology and quantity of cell were observed under a phase‐contrast microscope (black arrow: PSCs in high magnification [×200]). (B) These PSCs are stained by Oil Red O which showed the state with accumulated lipid droplets (blue arrow: lipid droplets stained by Oil Red O in high magnification [×200]). (C, D, E) Staining of NGF using immunofluorescence technique (×200). NGF is mainly localized to the cytoplasm of PSCs by red fluorescence (C), DAPI for nuclei (D) and the merged images (E)

To explore the role of PSCs in the development of PC, primary PSCs were isolated from human pancreas (Figure 1A). These activated PSCs were characteristic stained by Oil Red O which showed the state with accumulated lipid droplets (Figure 1B). And immunofluorescence showed that NGF was mainly localized to the cytoplasm of PSCs (Figure 1C,D,E). In summary, we confirmed that NGF was highly expressed in PSCs, which hinted its role in PC progression.

### 3.2 PSCs promote PC cell proliferation and invasion in PSCs‐PC cells co‐culture system

**Figure 2:**
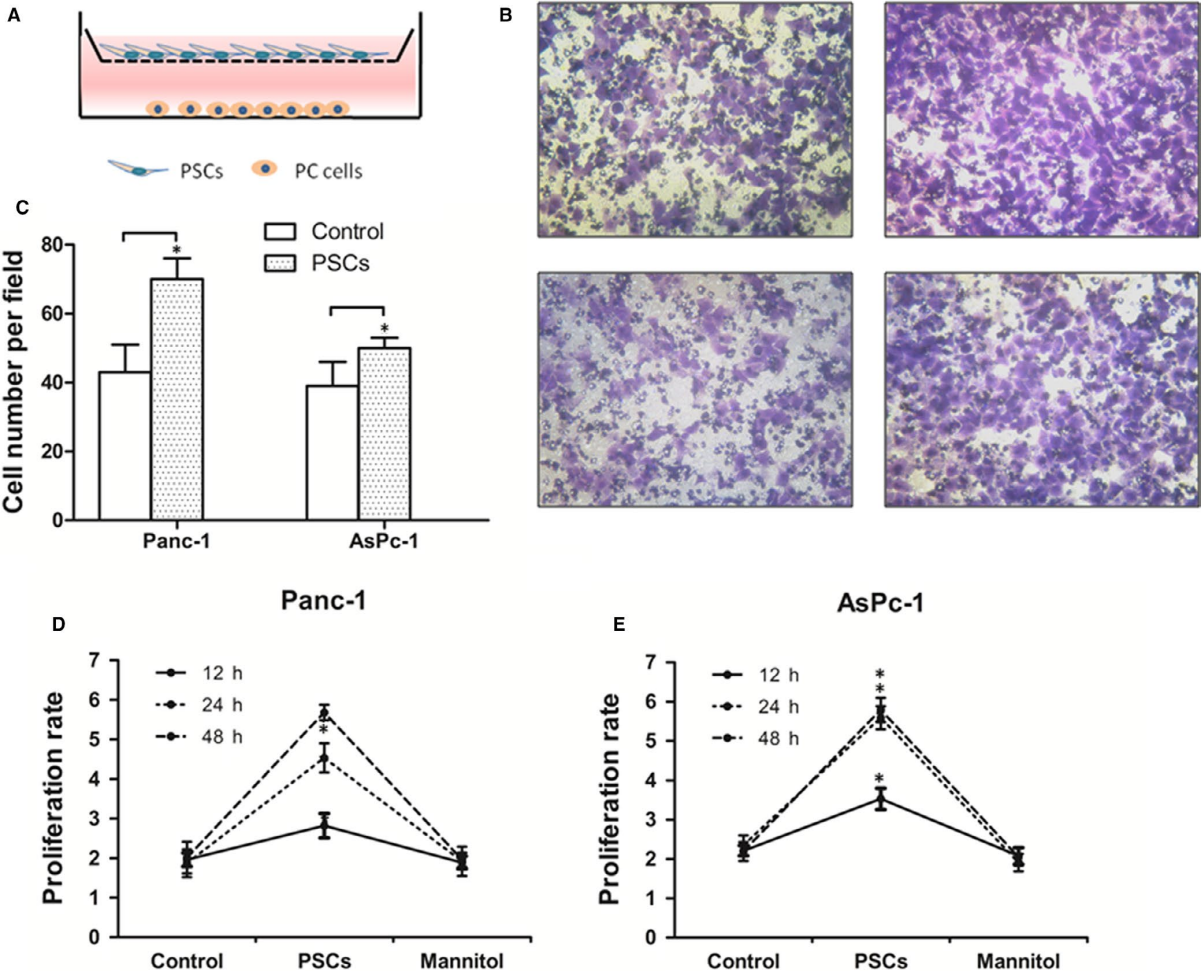
PSCs promote invasion and increase the proliferation of PC cells. (A) The PC cells and PSCs were directly co‐cultured in a no‐touch manner. Pancreatic cancer cell lines Panc‐1 or AsPc‐1 were placed in the lower chamber with or without PSCs in the upper chambers. (B) The invasion ability of Panc‐1 (the above listed) and AsPc‐1 (the below listed) cells was visibly increased when they co‐cultured with PSCs (the right side) compared with control group (the left side). (C) The migrated cancer cell numbers were visibly increased in the co‐cultured with PSCs in Panc‐1 and AsPc‐1 cells. (D, E) The proliferation rate of PC cells is significantly increased in the groups with PSCs co‐cultured, and the mannitol was a control for excluding influences of interstitial pressure. *P < .05 compared with control. All data from three independent experiments were analysed

To determine the effects of PSCs on PC cells, we used Transwell chamber with Matrigel coating to build a model which we called PSCs‐PC cells co‐culture system. PC cell lines Panc‐1 or AsPc‐1 were placed in the lower chamber with or without PSCs in the upper chambers (Figure 2A). The results showed that the invasion ability of PC cells (Panc‐1 and AsPc‐1) was visibly increased when they co‐cultured with PSCs (Figure 2B, C) (*P* < .05). We observed and calculated cell proliferation rate after 12, 24 and 48 hours, and the proliferation rate of PC cells was significantly increased in the groups with PSCs co‐cultured (Figure 2D,E) (*P* < .05). And, mannitol was a control for excluding influences of interstitial pressure. These results all showed that PSCs co‐cultured with PC cells could promote invasion and proliferation ability for PC cells.

### 3.3 Expressions of NGF and TrkA are increased in PSCs‐PC cells co‐culture system

**Figure 3:**
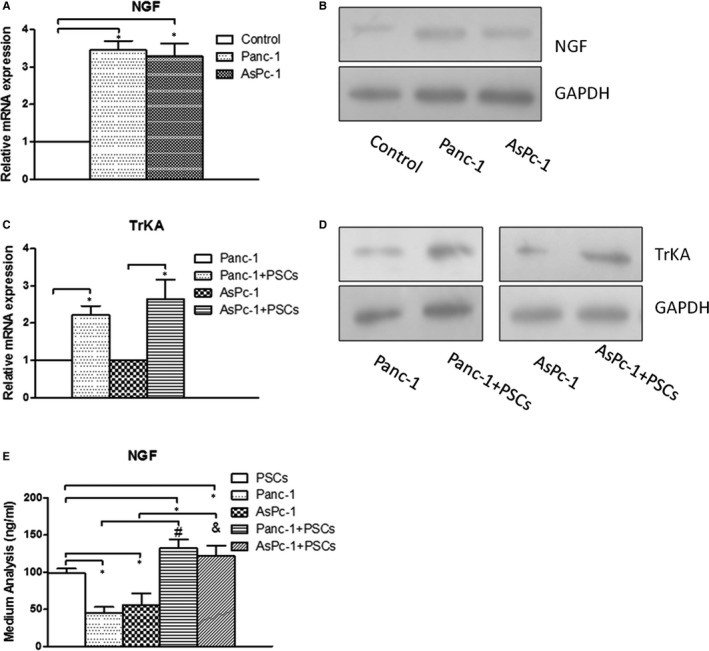
Co‐culture of PSCs and PC cells increased the expressions of NGF and TrkA. (A, B) The mRNA and protein expressions of NGF in PSCs were increased when co‐cultured with PC cells (Panc‐1 and AsPc‐1) than single PSCs. (C, D) The mRNA and protein expressions of TrkA in Panc‐1 and AsPc‐1 cells were increased in the co‐cultured system than single PC cells. (E) The NGF level in medium was increased highly compared with single cultivation by ELISA in co‐cultured system. *P < .05 compared with control. *^#^P *< .05 between PSCs‐Panc‐1 co‐cultured and Panc‐1 cells. ^&^ *P* < .05 between PSCs‐AsPc‐1 co‐cultured and AsPc‐1 cells. All data from three independent experiments were analysed

To assess the expression levels of NGF and TrkA, the RT‐PCR, ELISA and Western blot analysis were conducted to compare the mRNA and protein expression (Figure 3). We found that NGF expression level was increased in PSCs when co‐cultured with PC cells (Panc‐1 and AsPc‐1) than in PSCs when cultured alone for 24 hours (Figure 3A,B) (*P* < .05). And similarly, the TrkA expression level is significantly increased in PC cells co‐cultured with PSCs than in PC cells when cultured alone (Figure 3C,D) (*P* < .05). Next, we performed ELISA to test whether the expression level of NGF shows the similar result in medium. When PC cells co‐cultured with PSCs, the NGF level in medium increased obviously compared with any single cultivation (Figure 3E) (*P* < .05). Taken together, these findings indicate that PSCs may produce more NGF binding TrkA on PC cell membrane in PSCs‐PC cells co‐culture system.

### 3.4 NGF/TrkA axis plays a role in proliferation and invasion of PC cells in co‐culture system

**Figure 4:**
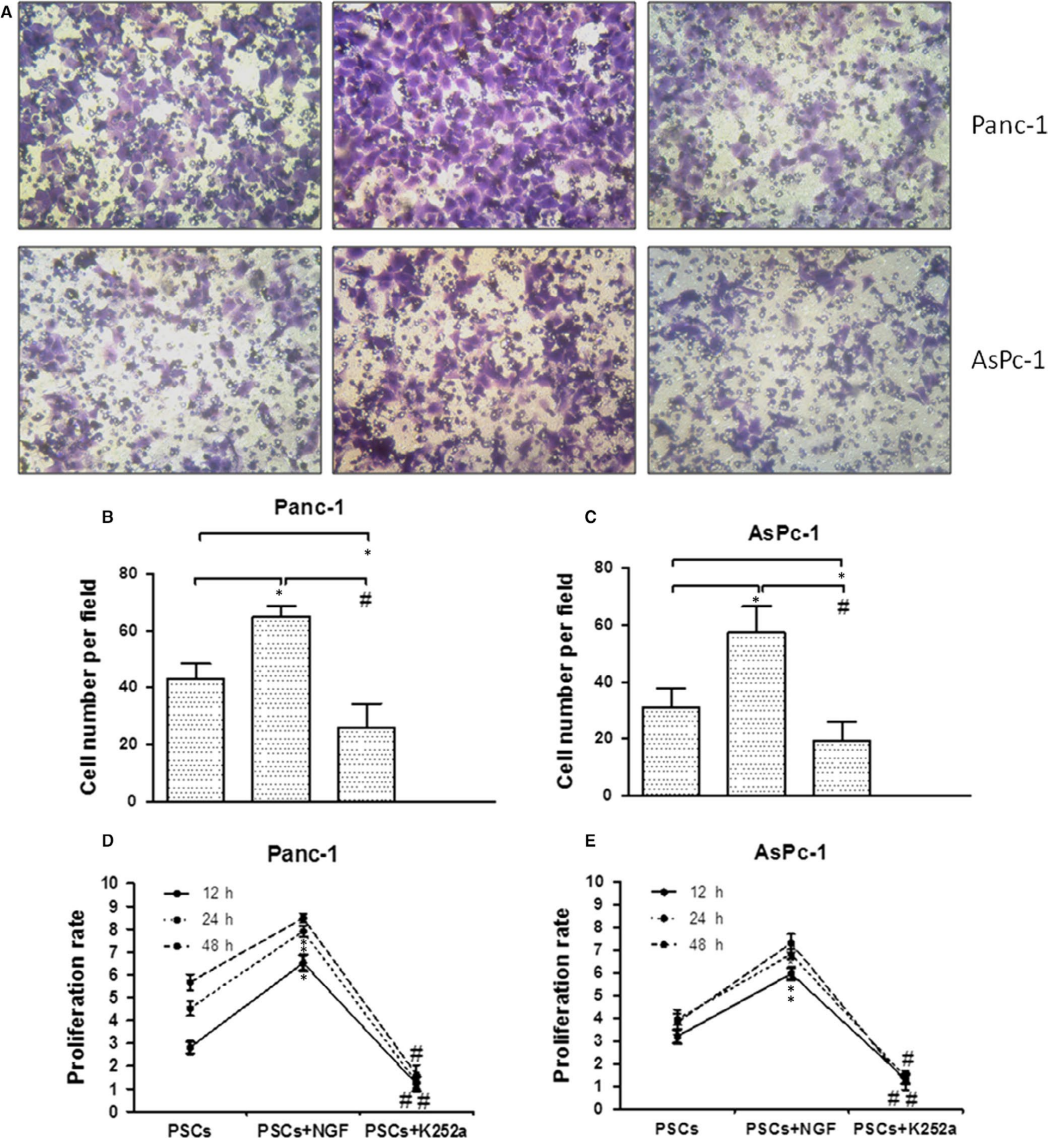
NGF/TrkA promoted the proliferation and invasion of PC cells in co‐culture system. (A, B, C) The invasion ability of Panc‐1 (the above listed) and AsPc‐1 (the below listed) cells was visibly affected by different treatments in the co‐cultured with PSCs: NGF (the middle) increased the invasion ability, but the K252a (the right) treatment significantly inhibited PC cell invasion compared with control (the left). (D, E) The proliferation rate of PC cells is significantly increased by NGF and inhibited by K252a in the co‐cultured with PSCs at different times. *P < .05 compared with control. *^#^P *< .05 between NGF group and K252a group. All data from three independent experiments were analysed

To further investigate the role of NGF/TrkA axis in the interaction between PSCs and PC cells, the synthetic NGF and K252a (inhibitor of TrkA) were used in the co‐culture system (Figure 4). The results demonstrated that the invasion ability in co‐culture system was enhanced by NGF (100 ng/mL) compared with control (*P* < .05), and the K252a treatment significantly inhibited PC cell invasion (Figure 4A‐C) (*P* < .05). The proliferation rate of Panc‐1 and AsPc‐1 cells in the co‐culture system with extra NGF was significantly increased compared with the control system. Also, K252a inhibited the proliferation rate of Panc‐1 and AsPc‐1 cells in PSCs‐PC cells co‐culture system (Figure 4D,E). These effects suggest that NGF/TrkA axis plays an important role in PC cell growth in co‐culture system.

### 3.5 The expression levels of the invasion‐related genes are influenced by si‐TrkA in co‐culture system

**Figure 5:**
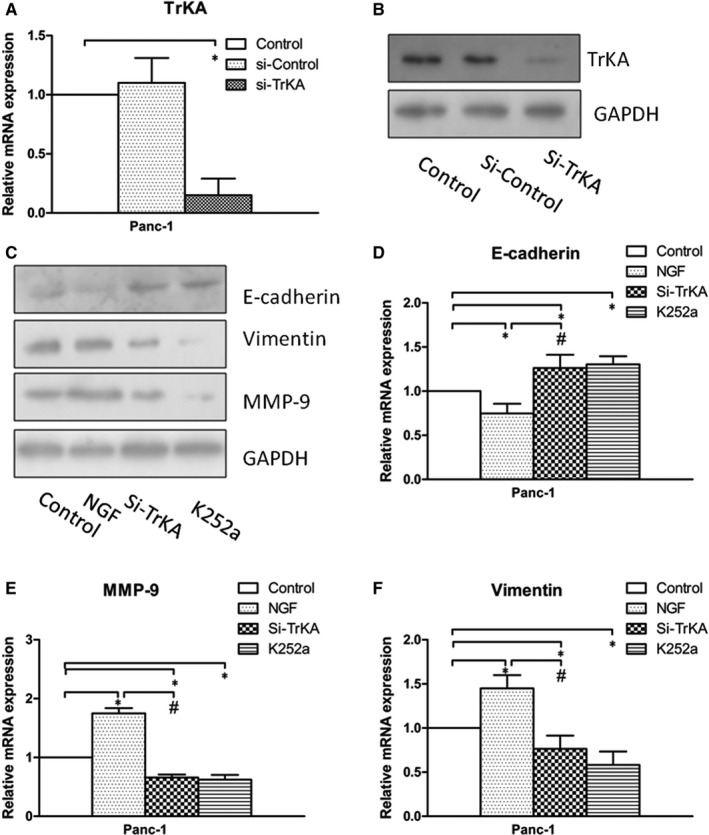
EMT process is mediated by NGF/TrkA in co‐culture system. (A, B) The transfection efficiencies were verified by Western blotting and real‐time PCR. Panc‐1 cells were transfected with TrkA siRNA. (C) The expression levels of MMP‐9, vimentin and E‐cadherin were changed by si‐TrkA and NGF shown by Western blot. (D) The E‐cadherin expression was increased by si‐TrkA or K252a but decreased by NGF in co‐culture system. (E, F) The expression of vimentin and MMP‐9 was decreased by si‐TrkA or K252a but increased obviously by NGF in co‐culture system. *P < .05 compared with control. *^#^P *< .05 between NGF group and si‐TrkA group. All data from three independent experiments were analysed

To determine the effects of NGF/TrkA signalling on cell invasion, Panc‐1 cells transfected with TrkA siRNA (si‐TrkA) were indirectly co‐cultured with PSCs (Figure 5). The TrkA expression had a obviously reduced trend in Panc‐1 cells which were transfected with Si‐TrkA in the level of mRNA and protein (Figure 5A,B) (*P* < .05). The Western blot results showed that the expression levels of MMP‐9, vimentin and E‐cadherin were changed by si‐TrkA (Figure 5C). It has shown that PSCs induce PC cell migration which is associated with EMT. What's more, the co‐culture system with NGF (100 ng/mL) showed the low level of E‐cadherin and high level of vimentin and MMP‐9 compared with the control group. The expression level of E‐cadherin was increased in co‐culture system with si‐TrkA or K252a (Figure 5D), and Si‐TrkA and K252a decreased the expression levels of vimentin and MMP‐9 obviously (Figure 5E,F) (*P* < .05). These data indicate that the activation of NGF/TrkA signalling enhanced the invasive ability of PC cells through influence of the expression of MMP‐9, vimentin and E‐cadherin.

### 3.6 NGF/TrkA signalling regulates EMT of PC cells via PI3K/AKT/GSK

**Figure 6:**
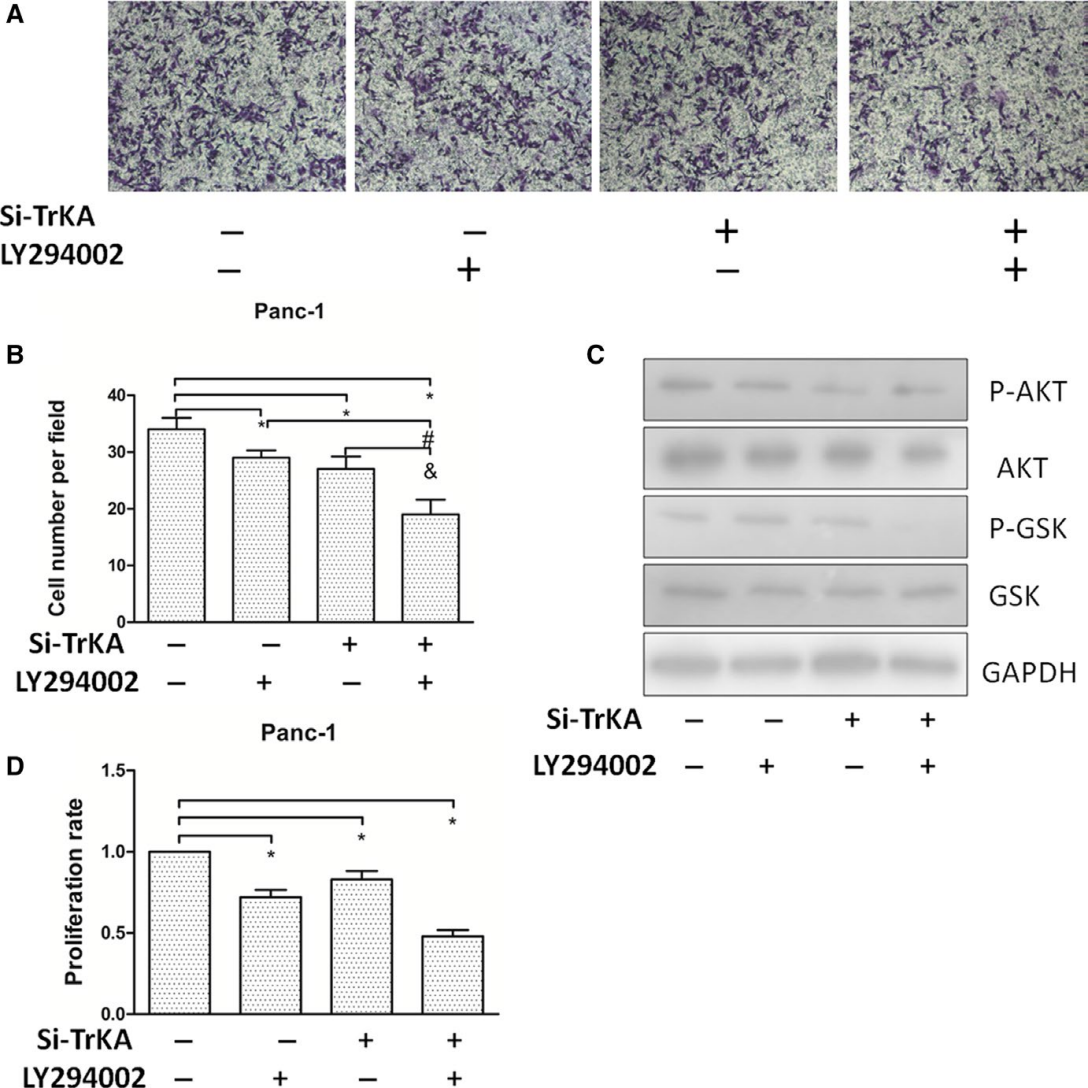
EMT of PC cells regulated by NGF requires activation of PI3K/AKT/GSK. (A, B) The invasion ability of Panc‐1 cells was visibly affected by different treatments with si‐TrkA and/or LY294002 in the co‐cultured with PSCs. The results showed that the invasion capacity of Panc‐1 cells was decreased with si‐TrkA or LY294002 treatment, especially both of combination. (C) The expression levels of PI3K signalling were evaluated by si‐TrkA and/or LY294002 shown by Western blot. (D) The expressions of p‐AKT and p‐GSK decreased visibly in Panc‐1 cells treated with LY294002 and/or si‐TrkA especially both of combination. *P < .05 compared with control. *^#^P *< .05 between LY294002 group and both of combination. ^&^ *P* < .05 between si‐TrkA group and both of combination. All data from three independent experiments were analysed

To further determine the mechanism of NGF/TrkA for the proliferation and invasion of PC, we evaluated PI3K signalling and its downstream targets, AKT and GSK, treated with si‐TrkA and LY294002 (an inhibitor of PI3K) (Figure 6). The results showed that the invasion capacity of Panc‐1 cells was decreased with si‐TrkA or LY294002 treatment, especially in the group of both combination (Figure 6A,B) (*P* < .05). Western blot assays showed that expressions of p‐AKT and p‐GSK decreased visibly in Panc‐1 cells treated with LY294002 and si‐TrkA (Figure 6C). Similarly, the proliferation of Panc‐1 cells was decreased with si‐TrkA and/or LY294002 treatment (Figure 6D) (*P* < .05). These data demonstrate that the abrogation of NGF/TrkA signalling could inhibit tumour growth and invasion by PI3K/AKT/GSK.

## 4 DISCUSSION

PC is one of the leading causes of cancer death among solid cancers.^1^ In fact, the high potential metastasis and rapid growth may be the main cause contributing to its high mortality rate.^17^ Recent years, tumour microenvironment is the research focus of cancer progression, and PSCs are involved in PC progression as the functional components.^6^, ^7^, ^18^, ^19^, ^20^ NGF and its receptors (P75 and TrkA) were always shown to be the acceleration role for the cancer development.^21^ Here, the work presented identifies that NGF and the PI3K/AKT signal pathway were involved in the mechanisms of interaction between PC and PSCs, promoting the proliferation and EMT in PC.

The pancreatic tumour microenvironment contains several cellular components, including the PC cells and PSCs,^18^, ^22^ which support tumour growth and progression. To clarify the interaction between both of them, the co‐culture system of PC cells and PSCs was used in this study. Based on our previous work, we propose a model in which the PC cells and PSCs were directly co‐cultured in a no‐touch manner.^23^ The advantage of this model is that the system could give consideration to the co‐culture which signify the microenvironment and also make it easier to analyse the action of PC cells and PSCs separately.

In this study, we showed that NGF was localized in PSCs (Figure 1), which suggests that the NGF secretion from PSCs has the work condition between action of PSCs and PC cells. Indeed, in the present co‐cultured model, the invasion ability and proliferation of PC cells (Panc‐1 and AsPc‐1) were visibly increased when they co‐cultured with PSCs compared with single cultivation (Figure 2). This finding suggests the critical function of PSCs on PC malignant progression in tumour microenvironment. Next based on this experimental system, we demonstrated an anxoaction role of PSCs on expression or secretion of NGF and an increasing expression of its receptor TrkA in the co‐culture system (Figure 3). Therefore, both NGF and TrkA were shown to be highly expressed in PSCs and PC cells, indicating their critical roles in PC invasion ability and proliferation.^24^ Usually, NGF has two main cell membrane receptors: TrkA and p75NTR. TrkA signalling has been widely found in cancer cells and exhibited specificity in NGF binding; thus, we chose the NGF/TrkA signalling as the main axis in the present study. In this study, we demonstrated that NGF enhanced the proliferation and invasion ability in co‐culture system, but the K252a treatment significantly inhibited both of them (Figure 4). Nevertheless, we believe that the activation of NGF/TrkA signalling plays only partial role in acceleration of PSCs on PC cells and other signalling pathways should be explored in the future research.

We also show in this study that PSC treatment increased the expression of both NGF and its receptor within the co‐culture system leading to increased invasiveness and proliferation. This finding suggests that the molecular function of NGF binding TrkA offers partly acceleration, and the high expression and activation of TrkA make contributions in the course of PSCs resulting in cell growth and invasiveness of PC cells. These findings provide the evidence that the pancreatic tumour microenvironment is a heterogeneous ecology and the interaction between cancer cells and stromal elements is complex and hard to make certain.^25^, ^26^ So, the deeper mechanism of action should be continued in the further investigation.

The evidence has come out that induction of the EMT programme is required for invasion.^27^, ^28^ The expression of cellular EMT marker changes, including MMP‐9, vimentin and E‐cadherin, has implications for the cancer invasion‐metastasis process. In this study, we demonstrated that some of EMT markers are regulated depending on the PC cells stimulated by si‐TrkA, leading to decreased invasiveness (Figure 5). Indeed, TrkA signalling has been widely found in cancer cells, exhibiting specificity in NGF binding. And, NGF binds TrkA to induce phosphorylation of TrkA for activation of intracellular signalling pathways (MAPK, PI3K and so on), resulting in cancer progression. Therefore, in the present study, we detected the expression of the PI3K/AKT/GSK signal after knockdown of TrkA (Figure 6). The results showed that the invasion capacity and proliferation of Panc‐1 cells were decreased with si‐TrkA or LY294002 treatment. And then, the expressions of p‐AKT and p‐GSK decreased visibly in Panc‐1 cells treated with LY294002 and si‐TrkA. Thus, we suggested that activation of PI3K/AKT/GSK signal might induce EMT programme and cell growth in the co‐culture system of PSCs and PC cells through NGF/TrkA. In consideration of the complexity and variety of signal pathways involved in the tumour microenvironment, the mechanism underlying NGF/TrkA function on EMT and proliferation between PSCs and PC cells still needs further study.

However, although we detected the regulation of NGF/TrkA in the co‐culture system of PSCs and PC cells in vitro, potentially, other factors or non‐canonical pathway may be excluded in the PC process promoted by PSCs.^11^, ^29^, ^30^ Further studies will be needed to investigate the mechanisms in animal experiments because of its simulation with the similar tumour microenvironment.

In conclusion, the present study showed that PC cells co‐cultured with PSCs can promote invasion and proliferation ability in PC cells. What's more, NGF/TrkA promoted the PC cell proliferation and invasion in the co‐culture system of PSCs and PC cells through the activation of the PI3K/AKT/GSK signal cascade. In the balance function of tumour microenvironment, we propose that NGF/TrkA could serve as an effective and potential therapeutic target for PC patients.

## CONFLICT OF INTEREST

The authors confirm that there are no conflicts of interest.

## AUTHOR CONTRIBUTIONS

LH and ZW designed the study. LH, ZW and JJ wrote the manuscript. JJ and JB performed analysis. LH, TQ, JB and JJ contributed to methodology. All authors read and approved the final manuscript.

## Data Availability

The data sets used and analysed during the current study are available from the corresponding author upon reasonable request.

## References

[1] Siegel RL , Miller KD , Jemal A . Cancer statistics, 2020. CA Cancer J Clin. 2020;70(1):7‐30. 31912902 10.3322/caac.21590

[2] Kleeff J , Korc M , Apte M , et al. Pancreatic cancer. Nature Rev Dis Prim. 2016;2:16022. 27158978 10.1038/nrdp.2016.22

[3] Alrawashdeh W , Jones R , Dumartin L , et al. Perineural invasion in pancreatic cancer: proteomic analysis and in vitro modelling. Mol Oncol. 2019;13:1075‐1091. 30690892 10.1002/1878-0261.12463 PMC6487729

[4] Kibe S , Ohuchida K , Ando Y , et al. Cancer‐associated acinar‐to‐ductal metaplasia within the invasive front of pancreatic cancer contributes to local invasion. Cancer Lett. 2019;444:70‐81. 30590101 10.1016/j.canlet.2018.12.005

[5] Li C , Cui L , Yang L , et al. Pancreatic stellate cells promote tumor progression by promoting an immunosuppressive microenvironment in murine models of pancreatic cancer. Pancreas. 2020;49:120‐127. 31856087 10.1097/MPA.0000000000001464

[6] Ren B , Cui M , Yang G , et al. Tumor microenvironment participates in metastasis of pancreatic cancer. Mol Cancer. 2018;17:108. 30060755 10.1186/s12943-018-0858-1 PMC6065152

[7] Takahashi K , Ehata S , Koinuma D , et al. Pancreatic tumor microenvironment confers highly malignant properties on pancreatic cancer cells. Oncogene. 2018;37:2757‐2772. 29511349 10.1038/s41388-018-0144-0 PMC5966364

[8] Koikawa K , Ohuchida K , Takesue S , et al. Pancreatic stellate cells reorganize matrix components and lead pancreatic cancer invasion via the function of Endo180. Cancer Lett. 2018;412:143‐154. 29061505 10.1016/j.canlet.2017.10.010

[9] Ligorio M , Sil S , Malagon‐Lopez J , et al. Stromal microenvironment shapes the intratumoral architecture of pancreatic cancer. Cell. 2019;178(1):160‐175.e27. 31155233 10.1016/j.cell.2019.05.012 PMC6697165

[10] Koikawa K , Ohuchida K , Ando Y , et al. Basement membrane destruction by pancreatic stellate cells leads to local invasion in pancreatic ductal adenocarcinoma. Cancer Lett. 2018;425:65‐77. 29580808 10.1016/j.canlet.2018.03.031

[11] Tang D , Wu Q , Yuan Z , et al. Identification of key pathways and gene changes in primary pancreatic stellate cells after cross‐talk with pancreatic cancer cells (BXPC‐3) using bioinformatics analysis. Neoplasma. 2019;66:446‐458. 30784291 10.4149/neo_2018_180925N714

[12] Ceyhan GO , Schafer KH , Kerscher AG , et al. Nerve growth factor and artemin are paracrine mediators of pancreatic neuropathy in pancreatic adenocarcinoma. Ann Surg. 2010;251:923‐931. 20395845 10.1097/SLA.0b013e3181d974d4

[13] Zhu ZW , Friess H , Wang L , et al. Nerve growth factor exerts differential effects on the growth of human pancreatic cancer cells. Clin Cancer Res. 2001;7:105‐112. 11205897

[14] Chopin V , Lagadec C , Toillon RA , Le Bourhis X . Neurotrophin signaling in cancer stem cells. Cell Mol Life Sci. 2016;73:1859‐1870. 26883804 10.1007/s00018-016-2156-7 PMC11108437

[15] Alagesan B , Contino G , Guimaraes AR , et al. Combined MEK and PI3K inhibition in a mouse model of pancreatic cancer. Clin Cancer Res. 2015;21:396‐404. 25348516 10.1158/1078-0432.CCR-14-1591 PMC4447091

[16] Ciuffreda L , Del Curatolo A , Falcone I , et al. Lack of growth inhibitory synergism with combined MAPK/PI3K inhibition in preclinical models of pancreatic cancer. Ann Oncol. 2017;28:2896‐2898. 28666315 10.1093/annonc/mdx335

[17] The global, regional and national burden of pancreatic cancer and its attributable risk factors in 195 countries and territories, 1990–2017: a systematic analysis for the Global Burden of Disease Study 2017. Lancet Gastroenterol Hepatol. 2019;4:934‐947. 31648972 10.1016/S2468-1253(19)30347-4 PMC7026711

[18] Dougan SK . The pancreatic cancer microenvironment. Cancer J. 2017;23:321‐325. 29189327 10.1097/PPO.0000000000000288

[19] Apte MV , Xu Z , Pothula S , Goldstein D , Pirola RC , Wilson JS . Pancreatic cancer: The microenvironment needs attention too! Pancreatology. 2015;15:S32‐S38. 25845856 10.1016/j.pan.2015.02.013

[20] Apte M , Pirola RC , Wilson JS . Pancreatic stellate cell: physiologic role, role in fibrosis and cancer. Curr Opin Gastroenterol. 2015;31:416‐423. 26125317 10.1097/MOG.0000000000000196

[21] Dang C , Zhang Y , Ma Q , Shimahara Y . Expression of nerve growth factor receptors is correlated with progression and prognosis of human pancreatic cancer. J Gastroenterol Hepatol. 2006;21:850‐858. 16704535 10.1111/j.1440-1746.2006.04074.x

[22] Zhang YF , Zhou YZ , Zhang B , et al. Pancreatic cancer‐derived exosomes promoted pancreatic stellate cells recruitment by pancreatic cancer. J Cancer. 2019;10:4397‐4407. 31413760 10.7150/jca.27590 PMC6691697

[23] Han L , Ma J , Duan W , et al. Pancreatic stellate cells contribute pancreatic cancer pain via activation of sHH signaling pathway. Oncotarget. 2016;7:18146‐18158. 26934446 10.18632/oncotarget.7776 PMC4951278

[24] Lin C , Ren Z , Yang X , et al. Nerve growth factor (NGF)‐TrkA axis in head and neck squamous cell carcinoma triggers EMT and confers resistance to the EGFR inhibitor erlotinib. Cancer Lett. 2020;472:81‐96. 31838083 10.1016/j.canlet.2019.12.015

[25] Thomas D , Radhakrishnan P . Tumor‐stromal crosstalk in pancreatic cancer and tissue fibrosis. Mol Cancer. 2019;18:14. 30665410 10.1186/s12943-018-0927-5 PMC6341551

[26] Rucki AA , Foley K , Zhang P , et al. Heterogeneous stromal signaling within the tumor microenvironment controls the metastasis of pancreatic cancer. Can Res. 2017;77:41‐52. 10.1158/0008-5472.CAN-16-1383 PMC5245778 27821486

[27] Gaianigo N , Melisi D , Carbone C . EMT and treatment resistance in pancreatic cancer. Cancers. 2017;9(12):122. 10.3390/cancers9090122 PMC5615337 28895920

[28] Aiello NM , Brabletz T , Kang Y , Nieto MA , Weinberg RA , Stanger BZ . Upholding a role for EMT in pancreatic cancer metastasis. Nature. 2017;547:E7‐E8. 28682339 10.1038/nature22963 PMC5830071

[29] Allam A , Thomsen AR , Gothwal M , Saha D , Maurer J , Brunner TB . Pancreatic stellate cells in pancreatic cancer: In focus. Pancreatology. 2017;17:514‐522. 28601475 10.1016/j.pan.2017.05.390

[30] Roy I , Boyle KA , et al. Cancer cell chemokines direct chemotaxis of activated stellate cells in pancreatic ductal adenocarcinoma. Lab Invest 2017;97 :302‐317. 28092365 10.1038/labinvest.2016.146 PMC5334280

